# Olive pomace oil and acid oil as alternative fat sources in growing-finishing broiler chicken diets

**DOI:** 10.1016/j.psj.2022.102079

**Published:** 2022-07-26

**Authors:** G. Verge-Mèrida, D. Solà-Oriol, A. Tres, M. Verdú, G. Farré, C. Garcés-Narro, A.C. Barroeta

**Affiliations:** ⁎ Animal Nutrition and Welfare Service, Department of Animal and Food Sciences, Universitat Autònoma de Barcelona, 08193 Bellaterra, Spain; † Nutrition, Food Science and Gastronomy Department INSA-XIA, Universitat de Barcelona, 08921 Santa Coloma de Gramenet, Spain; ‡ Department of Animal Nutrition and Feed Industry*,* bonÀrea Agrupa, 25210 Guissona, Spain; § Department of Animal Production and Health, Universidad CEU Cardenal Herrera, CEU Universities, 46115 Alfara de Patriarca, Valencia, Spain

**Keywords:** olive pomace oil, acid oil, digestibility, fatty acid, by-product

## Abstract

The aim of the present study was to investigate the effect of dietary supplementation of olive pomace oil and olive pomace acid oil, which are rich in monounsaturated fatty acids (**FA**) but differ in free FA content, on growth performance, digestibility and FA profile of abdominal fat and breast meat. A total of 3,048 one-day-old mixed-sex broiler chickens (Ross 308) were randomly distributed into 24 pens and 3 dietary treatments (8 replicates per treatment). Experimental diets were administered for growing (from 22 to 29 d) and finishing (from 30 to 39 d) periods, consisting of a basal diet supplemented with 6% (as-fed basis) palm oil (**PO**), olive pomace oil (**O**), or olive pomace acid oil (**OA**). Animals fed O achieved the lowest feed conversion ratio (*P* < 0.01), together with the highest AME value (*P* = 0.003), but no differences were observed between OA and PO. Regarding FA digestibility, O and OA showed higher values than PO for all FA in both apparent ileal digestibility (**AID**) and apparent total tract digestibility. Comparing the AID between O and OA, no differences were observed for total FA, monounsaturated FA, or polyunsaturated FA, but animals fed OA showed lower AID values for saturated FA than those fed O (*P* < 0.001). The FA profile of abdominal fat and breast meat reflected that of the diet, with higher monounsaturated FA and lower saturated FA in animals fed O and OA compared to those fed PO. In sum, the inclusion of both olive pomace oil and acid oil in growing-finishing broiler chicken diets led to great performance parameters and high FA digestibility values, together with an enrichment with monounsaturated FA in abdominal fat and breast meat compared to the use of palm oil. However, a better AID of saturated FA and feed conversion ratio is achieved with O compared to OA.

## INTRODUCTION

Fats and oils are widely included in broiler chicken diets due to their high energy content and supply of essential fatty acids (**FA**). However, the need for more efficient and sustainable animal production necessitates a continuous search for alternative fat sources to achieve these goals. In this regard, olive pomace oil and olive pomace acid oil could be 2 potential alternative fat sources for use in broiler chicken diets. Both are rich in monounsaturated FA (**MUFA**), and particularly in oleic acid (C18:1 n-9), which is of special interest since unsaturated FA (**UFA**) are known to be better absorbed than saturated FA (**SFA**; Ravindran et al., 2016). Moreover, the inclusion of olive-derived oils in broiler chicken diets could enrich the FA profile in meat and deposition fat with UFA (Skřivan et al., 2018).

In terms of molecular structure, olive pomace oil is mainly composed of triacylglycerols, while olive pomace acid oil, a by-product generated from the chemical refining of olive pomace oil, accumulates a high content of free FA (**FFA**, 40–60%; Varona et al., 2021a). There are controversial results in the literature regarding the effects of dietary FFA content on fat utilization and AME. Some authors have reported a decrease in the AME value and lower digestibility values when dietary FFA content increases (Wiseman and Salvador, 1991; Blanch et al., 1995,^1996^; Wiseman et al., 1998), while other studies observed no negative impact on performance or feed efficiency (Zumbado et al., 1994; Vilarrasa et al., 2015; Rodriguez-Sanchez et al., 2019a,^2021^; Jimenez-Moya et al., 2021a). In this regard, and according to the results of previous studies, it may be better to use acid oils from unsaturated sources and include them in growing-finishing broiler chicken diets rather than in earlier stages, as age has a positive effect on FFA absorption and fat utilization (Tancharoenrat et al. 2013; Roll et al. 2018; Rodriguez-Sanchez, et al. 2019b; Viñado et al. 2019; Jimenez-Moya et al. 2021a). Additionally, the inclusion of olive pomace acid oil, a food-chain by-product, could help to reduce feeding costs as it is usually competitively priced, and its direct application might contribute to a circular bioeconomy system and more sustainable production compared to other uses of these by-products that require further processing. Therefore, olive pomace oil and acid oil, which are rich in MUFA but differ in FFA content, could be suggested as feeding fats for growing-finishing broilers. However, before recommending them, it is essential to evaluate their effects to assure suitable animal production performance, especially under commercial production practices.

Therefore, the aim of the present study was to investigate the potential use of olive pomace oil and olive pomace acid oil as fat sources in growing-finishing broiler diets. This was assessed by studying the effect of dietary supplementation of olive pomace oil and olive pomace acid oil on the growth performance, carcass parameters, digestibility, abdominal fat deposition, and FA profile of abdominal fat and breast meat.

## MATERIALS AND METHODS

### Experimental Fats

The chemical composition of experimental fats is shown in Table 1. Olive pomace oil and olive pomace acid oil were supplied by RIOSA S.A. (Jaén, Spain), and palm oil was provided by bonÀrea Agrupa (Guissona, Spain). All samples were analyzed in duplicate for FA composition, lipid class composition, moisture, impurities, and unsaponifiable matter as described by Varona et al. (2021b).

**Table 1:** Composition of experimental fats included in the grower and finisher diets of broiler chickens.

	Experimental fats		
Item^1^	POO	OO	OAO
Fatty acid composition, %			
C 14:0	0.96	0.03	0.06
C 16:0	42.56	12.66	12.43
C16:1 n-7	0.16	0.91	1.04
C 18:0	4.53	2.69	2.69
C18:1 n-9	40.80	70.06	63.24
C18:1 n7	0.61	1.59	1.68
C18:1 trans	0.02	0.12	0.43
C18:2 n-6	9.50	10.09	15.83
C18:3 n-3	0.30	0.68	0.90
C20:0	0.40	0.48	0.52
C20:1 n-9	0.15	0.35	0.28
C22:0	-	0.23	0.46
C24:0	-	0.11	0.44
SFA	48.61	16.20	16.60
MUFA	41.59	73.03	66.67
PUFA	9.80	10.77	16.73
UFA:SFA	1.06	5.17	5.02
n-6:n-3	31.89	14.73	17.61
Lipid class composition, %			
TAG	87.98	91.09	24.47
DAG	8.67	8.38	18.60
MAG	0.05	0.32	2.34
FFA	3.31	0.21	54.59
MIU, %			
Moisture	-	-	0.73
Impurities	0.49	0.28	1.37
Unsaponifiable	0.22	1.44	4.53

Abbreviations: POO, palm oil; OO = olive pomace oil; OAO, olive pomace acid oil; SFA, saturated fatty acids; MUFA, monounsaturated fatty acids; PUFA, polyunsaturated fatty acids; UFA, unsaturated fatty acids; TAG, triacylglycerols; DAG, diacylglycerols; MAG, monoacylglycerols; FFA, free fatty acids; MIU, moisture, impurities and unsaponifiable.

1 All samples were analyzed in duplicate.

### Experimental Design and Diets

The study was conducted on the experimental facilities of bonÀrea Agrupa (Nial Farm, Guissona, Spain). All animal housing and husbandry was in accordance with the European Union Guidelines (2010/63/EU), and all management practices and procedures were approved by the Animal Ethics Committee (**CEEAH**) of the Universitat Autònoma de Barcelona (code number: 3938). A total of 3,048 newly hatched mixed-sex broiler chickens (Ross 308; 40.9 ± 0.26 g of BW, mean ± SD) were obtained from the commercial hatchery of bonÀrea Agrupa. On arrival, birds were distributed into 24-floor pens (12 m^2^, 127 animals per pen), balanced by body weight and assigned to 1 of the 3 dietary treatments (8 replicates per treatment). Each pen was provided with feed and water that could be accessed ad libitum throughout the study. Environmental conditions were automatically controlled, following the recommendations and specifications of the Ross 308 management handbook (Aviagen, 2018).

A 4-phase feeding program was used, consisting of a pre-starter diet from 0 to 7 d, a starter diet from 8 to 21 d, a grower diet from 22 to 29 d and a finisher diet from 30 to 39 d, all in pelleted form. The ingredients of the experimental diets are shown in Table 2, and these were formulated to meet or exceed requirements (FEDNA, 2018). Pre-starter and starter diets were common to all animals. For grower and finisher diets, 3 experimental treatments were obtained as the result of adding 6% (as-fed basis) of different fat sources: palm oil (**PO**), olive pomace oil (**O**), and olive pomace acid oil (**OA**). Silicate (Ibersil D-100M; IQESIL S.A., Zaragoza, Spain) was added to the finisher diets (1.00% as-fed basis) to increase the amount of HCl-insoluble ash as an inert digestibility marker.

**Table 2:** Ingredient composition of the pre-starter, starter, grower and finisher diets (as-fed basis) for broiler chickens.

	Common period		Experimental period	
Ingredients, %	Pre-Starter	Starter	Grower	Finisher
Corn	24.01	35.07	35.11	34.99
Soybean meal 47%	36.20	30.38	23.79	19.74
Wheat	30.04	24.86	10.85	15.01
Sorghum	-	-	10.00	10.00
Sunflower meal	-	-	10.00	10.00
Soybean oil	4.69	4.72	-	-
Experimental fat^1^	-	-	6.00	6.00
Silicate	1.00	1.00	1.00	1.00
Dicalcium phosphate	1.36	1.12	0.98	0.73
Calcium carbonate	1.04	1.12	0.69	1.02
Vit-Min. premix^2^	0.45	0.45	0.45	0.45
Sodium chloride	0.32	0.30	0.29	0.28
DL-Methionine^3^	0.36	0.34	0.28	0.24
L-Lysine^4^	0.39	0.46	0.46	0.45
L-Threonine	0.12	0.14	0.09	0.08
L-Valine	0.02	0.04	0.01	0.01

1 Palm oil, olive pomace oil or olive pomace acid oil.

2 Provides, per kg of feed: vitamin A (retinyl acetate), 10,000 IU; vitamin D, 4,700 IU; vitamin E (dl-alpha-tocopheryl acetate), 100 IU; vitamin K, 4 mg; vitamin B1, 4 mg; vitamin B2, 8 mg; vitamin B6, 5 mg; vitamin B12, 18 µg; biotin, 0.25 mg; Cu, 13.12 mg (from CuSO_4_); I, 1.25 mg (from KI); Mn, 121.5 mg (from MnO_2_); Se, 0.3 mg (from Na_2_SeO_3_); Zn, 67.5 mg (from ZnO); Fe, 141.75 mg (from FeSO_4_); phytase, 1,500 FYT (Ronozyme Trade mark; DSM, Herleen, The Netherlands).

3 DL-2-hydroxy-4-methylthiobutanoic acid (HMTBa), the hydroxyl analogue of DL-methionine.

4 L-Lysine sulphate.

### Controls and Sampling

Feed consumption and BW (pen-basis) were recorded at 7, 21, 29, and 39 d of age. This was used to calculate the ADG, the ADFI and the feed conversion ratio (**FCR**) for each period and for the overall study. Mortality was recorded and weighed to adjust and correct these parameters. The digestibility balance was determined from 30 to 36 d of age in a subset of 144 animals (n = 48 animals/treatment). Animals selected for the digestibility balance were closer to the average BW (mean ± 0.5 SD) of each sex within each pen (3 males and 3 females were selected from each pen). At d 36, excreta samples were collected from selected animals by abdominal-massage stimulation and then these animals were electrically stunned (Reference: 105523; FAF, Saint-Sernin-sur-Rance, France) and immediately exsanguinated to obtain ileal content. The ileal digestive contents (from the junction with Meckel's diverticulum to a point 1 cm proximal to the iliocecal junction) of samples from each sex group in each pen were pooled, homogenized, freeze dried (LyoAlpha 10/15; Telstar, Barcelona, Spain), ground (1 mm screen diameter) and kept at 5°C until further analyses. Animals euthanized for digestibility balance were adjusted as mortality for the performance parameters calculations.

At d 39, animals were fasted for 10 h and slaughtered at the bonÀrea Agrupa commercial slaughterhouse (Guissona, Spain). All carcasses were processed (blood, feathers, viscera, head, and feet were removed) and weighed to obtain the carcass yield. Breast meat and abdominal fat pad were obtained from the 5 female broilers per pen (n = 40 animals/treatment) that were closest to the average BW (mean ± 0.5 SD). Samples of breast meat were homogenized, minced, pooled for each pen, freeze-dried, ground (1-mm screen diameter), and kept at 5°C until further analyses. Samples of abdominal fat pad were homogenized, pooled for each pen and kept at −20°C until further analyses.

### Chemical Analyses

The composition of feeds is shown in Table 3. Analytical determinations of the feeds were performed according to AOAC International (2005) methods: dry matter (Method 934.01), ash (Method 942.05), crude protein (Method 954.01), ether extract (Method 920.39), and crude fiber (Method 962.09). The gross energy was determined with an adiabatic calorimeter (Parr 6300 Calorimeter, Parr Instrument Company, Moline, IL) according to the Standard UNE-EN ISO 9831:2004. Lipid class composition was analyzed by size exclusion HPLC with refractive index detection, following the method described by Varona et al. (2021b). The FA content of feed, ileal content and excreta were analyzed following the method described by Sukhija and Palmquist (1988). Abdominal fat pad and breast meat FA were analyzed following the method described by Carrapiso et al. (2000). Nonadecanoic acid (C19:0, Sigma Aldrich Chemical Co.; St. Louis, MO) was added as an internal standard. The FA composition of the final extract was injected in a gas chromatograph (HP6890, Agilent Technologies; Waldbronn, Germany) following the method conditions described by Cortinas et al. (2004). HCl-insoluble ash was determined in feeds, ileal content and feces according to the methods of European Commission Regulation n° 152/2009(“European Commission Regulation No 152/2009 of 27 January 2009. Laying down the methods of sampling and analysis for the official control of feed - Publications Office of the EU,”).

**Table 3:** Macronutrient and energy content, fatty acid, and lipid class composition of the diets fed to broiler chickens.

			Grower diets			Finisher diets		
Item^1^	Pre-starter diet	Starter diet	PO	O	OA	PO	O	OA
Macronutrient and energy content, %								
Dry matter	89.19	89.42	89.70	90.14	89.77	90.27	90.34	90.36
Crude protein	20.15	20.16	18.98	19.11	19.68	18.53	18.19	18.45
SID Methionine*	0.65	0.61	0.56	0.56	0.56	0.51	0.51	0.51
SID Lysine*	1.24	1.13	1.04	1.04	1.04	0.94	0.94	0.94
Ether extract	5.91	5.40	7.71	7.76	7.49	8.06	7.71	8.07
Crude fiber	3.24	3.09	4.47	4.48	4.98	5.06	4.43	4.6
Ash	6.13	5.45	5.38	5.17	5.46	5.62	5.54	5.81
Calcium*	1.08	1.04	0.85	0.85	0.85	0.90	0.90	0.90
Phosphorus*	0.63	0.56	0.60	0.60	0.60	0.54	0.54	0.54
Digestible phosphorus*	0.48	0.43	0.42	0.42	0.42	0.37	0.37	0.37
Chloride*	0.24	0.23	0.24	0.24	0.24	0.23	0.23	0.23
Gross energy, kcal/kg	4,059	4,162	4,231	4,243	4,203	4,232	4,221	4,225
Fatty acid composition, %								
C16:0	15.99	20.03	30.62	13.67	15.49	30.89	13.89	17.50
C18:0	3.75	3.73	3.87	2.81	3.25	3.81	2.82	3.34
C18:1 n-9	18.65	24.41	33.93	51.42	43.80	34.71	51.72	43.44
C18:1 n-7	1.35	1.22	1.06	1.91	1.67	1.06	1.56	1.83
C18:2 n-6	54.12	45.71	27.71	27.14	32.25	26.84	26.72	30.42
C18:3 n-3	5.46	4.20	1.32	1.51	1.68	1.19	1.42	1.50
Minor fatty acids	0.69	0.69	1.50	1.53	1.86	1.50	1.87	1.96
SFA	20.59	24.67	36.22	17.21	19.94	36.21	17.46	22.43
MUFA	19.83	25.42	34.75	54.14	46.13	35.75	54.40	45.64
PUFA	59.58	49.91	29.03	28.65	33.93	28.03	28.14	31.93
UFA:SFA	3.86	3.05	1.76	4.81	4.02	1.76	4.73	3.46
Lipid class composition, %								
TAG	58.93	70.04	82.54	84.14	54.84	84.21	84.98	56.38
DAG	15.85	12.18	8.83	9.17	13.19	8.27	8.84	12.69
MAG	1.99	1.14	0.43	0.58	1.27	0.45	0.13	1.10
FFA	23.23	16.64	8.19	6.11	30.70	7.08	0.18	29.83

Abbreviations: DAG, diacylglycerols; FFA, free fatty acids; MAG, monoacylglycerols; MUFA, monounsaturated fatty acids; O, olive pomace oil diet; OA, olive pomace acid oil diet; PO, palm oil diet; PUFA, polyunsaturated fatty acids; SFA, saturated fatty acids; TAG, triacylglycerols; UFA, unsaturated fatty acids.

⁎ Calculated values from the theoretical formulation of the diets

1 All samples were analyzed in duplicate.

### Calculations

The apparent digestibility of a particular FA (X) was calculated as follows:

%apparentdigestibilityofX={1−[(Xf/Mf)/(Xd/Md)]}×100

where Xf is the concentration of a particular FA in excreta or ileal content, Mf is the concentration of the inert marker in excreta or ileal content, Xd is the concentration of a particular FA in the diet, and Md is the concentration of the inert marker in the diet. The ileal digestible energy and AME of feeds was calculated from the product of energy apparent digestibility and its corresponding feed gross energy.

### Statistical Analysis

The normality of the data and homogeneity of variance were verified using the CAPABILITY procedure of SAS (version 9.4, SAS Inst. Inc.; Cary, NC). All data were analyzed using the GLM procedure of SAS. For performance, carcass parameters and FA profile of breast meat and abdominal fat pad, diet was defined as the main factor. For digestibility balance, diet and sex were defined as the main factors. No interactions were found between diet and sex for any of the variables studied. For all analyses, the experimental unit was the pen (n = 8 for each treatment), and differences between means were tested using Tukey's adjust correction for multiple comparisons. The results in the tables are reported as least square means. For all statistical analyses, significance was declared at *P* < 0.05 and tendencies were discussed at 0.05 < *P* < 0.10.

## RESULTS

### Characterization of Experimental Oils and Diets

The composition of the experimental oils is presented in Table 1. Regarding the FA composition, olive pomace oil, and olive pomace acid oil were rich in monounsaturated FA (73.03 and 66.67%, respectively), while palm oil was rich in saturated FA (48.45%). Of the experimental oils, olive pomace acid oil had the highest content of polyunsaturated FA (**PUFA**; 16.73%). The main FA was oleic acid in the cases of olive pomace oil and olive pomace acid oil (70.06 and 63.24%, respectively), while for palm oil, palmitic (42.56%), and oleic (40.80%) appeared in similar proportions. Both olive pomace oil and palm oil were composed mainly of triacylglycerols (∼ 90%), while olive pomace acid oil had a higher amount of free FA (54.59%). Additionally, the highest values for moisture, impurities, and unsaponifiable (**MIU**) were found for olive pomace acid oil (6.63%), while olive pomace (1.72%) and palm oil (0.71%) had lower values. The composition of experimental diets is shown in Tables 2 and 3. The gross energy and all macronutrient content values were similar among dietary treatments. The FA and lipid class composition mirrored that of the added experimental oils, the O and OA diets being richer in MUFA while PO was richer in SFA. Also, the OA diet showed a higher content in FFA.

### Growth Performance and Feed Intake

The effects of the dietary experimental oils on growth performance and feed intake are shown in Table 4. No differences were found between OA and PO in any performance parameter (*P* > 0.10). Considering the grower period, from d 22 to 29, animals fed O had a higher ADG than those fed OA (*P* = 0.017) and the lowest FCR among dietary treatments (*P* = 0.004). No differences were observed in BW at d 29 or in ADFI during this period. For the finishing period, from d 30 to 39, animals fed O had the lowest ADFI and FCR among dietary treatments (*P* < 0.05) and showed a tendency to reach a higher BW at d 39 than those fed OA. Concerning the entire production cycle, animals fed O had the lowest FCR among dietary treatments (*P =* 0.016) and a tendency to have a higher ADG than those fed OA (*P* = 0.071). No differences were found in carcass weight or carcass yield among dietary treatments (*P* > 0.10).

**Table 4:** Growth performance and carcass parameters of broiler chickens fed different dietary fat sources.

	Common diets				
Item^1^	Mean	SD			
From 0 to 21 d					
BW 0 days, g	40.85	0.26			
BW 7 days, g	181.88	3.33			
ADFI, g/d	59.0	1.82			
ADG, g/d	43.0	0.56			
FCR, g/g	1.370	0.04			

	Dietary treatments				
	PO	O	OA	SEM^2^	*P*-value
From 22 to 29 d					
BW 21 days, g	944.3	945.3	944.4	4.44	0.985
BW 29 days, g	1,679	1,692	1,670	7.73	0.161
ADFI, g/d	135.2	133.6	134.8	1.11	0.577
ADG, g/d	91.8^ab^	93.3^a^	90.7^b^	0.67	0.017
FCR, g/d	1.478^a^	1.432^b^	1.487^a^	0.012	0.004
From 30 to 39 d					
BW 39 days, g	2,674	2,701	2,647	15.68	0.071
ADFI, g/d	189.0^ab^	183.2^b^	191.5^a^	1.81	0.013
ADG, g/d	100.2	101.0	98.9	0.95	0.296
FCR, g/g	1.897^a^	1.815^b^	1.935^a^	0.019	< 0.001
From 0 to 39 d					
ADFI, g/d	112.6	110.6	111.8	0.99	0.385
ADG, g/d	67.5	68.2	66.8	0.40	0.071
FCR, g/g	1.668^a^	1.622^b^	1.673^a^	0.012	0.016
Carcass parameters					
Carcass weight, g	2,016	2,007	1,968	20.15	0.210
Carcass yield, %	75.42	74.29	73.72	0.61	0.154

Abbreviations: ADFI, average daily feed intake; ADG, average daily gain; BW, body weight; FCR, feed conversion ratio; O, olive pomace oil diet; OA, olive pomace acid oil diet; PO, palm oil diet; SEM, standard error of the mean.

1 Values of ADFI and ADG are expressed as-fed basis.

2 n = 8.

a-b Values within a row with different superscripts differ significantly at *P* < 0.05.

### Digestibility Balance

The ileal apparent digestible energy, AME and apparent ileal (**AID**), and total tract (**ATTD**) FA digestibility of the feeds are presented in Table 5. No effect of sex or interactions between diet and sex were detected for any of the variables studied and therefore, only diet effects are presented. For apparent ileal digestible energy and AME, O showed the highest values among dietary treatments, while no differences were observed between OA and PO (*P* < 0.01). Considering the digestibility of FA, O and OA showed higher values than PO for all analyzed FA in both AID and ATTD (*P* < 0.001). When comparing O and OA, no differences were observed for the AID of total FA, MUFA, and PUFA, but O had higher values for SFA than OA (*P* < 0.001). For ATTD, O showed higher values for total FA, SFA and MUFA than OA (*P* < 0.001), and no differences were observed for PUFA.

**Table 5:** Feed apparent digestible and metabolizable energy (kcal/kg) and fatty acid apparent ileal and total tract digestibility in 36-day-old broiler chickens fed different dietary fat sources.

	Dietary treatments				
Item	PO	O	OA	SEM^1^	*P*-value^2^
AID, %					
Apparent ileal digestible energy, kcal/kg	3,024.97^b^	3,288.03^a^	3,052.98^b^	45.96	0.003
Total FA	85.92^b^	95.75^a^	93.88^a^	0.64	< 0.001
SFA	71.74^c^	89.75^a^	84.75^b^	1.43	< 0.001
MUFA	92.61^b^	96.44^a^	95.25^a^	0.44	< 0.001
PUFA	95.88^b^	97.88^a^	97.81^a^	0.20	< 0.001
C16:0	72.82^b^	90.50^a^	86.31^a^	1.33	< 0.001
C18:0	66.04^c^	86.31^a^	79.94^b^	1.59	< 0.001
C18:1 n-9	93.27^b^	96.44^a^	95.31^a^	0.38	< 0.001
C18:1 n-7	89.37^b^	95.88^a^	94.44^a^	0.64	< 0.001
C18:2 n-6	95.67^b^	97.75^a^	97.69^a^	0.20	< 0.001
C18:3 n-3	97.73^b^	99.56^a^	100.00^a^	0.47	0.028
ATTD, %					
AME, kcal/kg	3,087.56^b^	3,281.07^a^	3,153.88^b^	39.25	0.003
Total FA	85.06^c^	95.25^a^	93.06^b^	0.47	< 0.001
SFA	70.69^c^	90.75^a^	85.59^b^	0.95	< 0.001
MUFA	92.25^c^	95.56^a^	93.81^b^	0.36	< 0.001
PUFA	93.94^b^	96.88^a^	97.19^a^	0.26	< 0.001
C16:0	71.81^c^	91.69^a^	87.25^b^	0.88	< 0.001
C18:0	63.00^c^	87.25^a^	79.44^b^	1.28	< 0.001
C18:1 n-9	92.25^c^	95.56^a^	93.81^b^	0.36	< 0.001
C18:1 n-7	88.63^c^	94.63^a^	92.06^b^	0.45	< 0.001
C18:2 n-6	94.13^b^	96.88^a^	97.19^a^	0.24	< 0.001
C18:3 n-3	93.00^b^	99.00^a^	99.75^a^	0.38	< 0.001

Abbreviations: AID , apparent ileal digestibility; ATTD, apparent total tract digestibility; MUFA, monounsaturated fatty acids; O, olive pomace oil diet; OA, olive pomace acid oil diet; PO, palm oil diet; PUFA, polyunsaturated fatty acids; SEM, standard error of the mean; SFA, saturated fatty acids.

1 n = 16 for each treatment (8 replicates × 2 sex).

2 No effect of sex or interactions between diet and sex were detected for any of the variables studied and therefore, only diet effects are presented.

a-c Values within a row with different superscripts differ significantly at *P* < 0.05.

### Fatty Acid Composition of Abdominal Fat Pad and Breast Meat

The FA composition of abdominal fat pad and breast meat are presented in Tables 6 and 7, respectively. The FA profiles of abdominal fat pad and breast meat resembled that of the diet. Animals fed O had a higher abdominal fat deposition than those fed OA (*P* = 0.023). In relation to abdominal fat pad, O had the highest MUFA content and the highest UFA:SFA ratio, together with the lowest PUFA content among dietary treatments (*P* < 0.01). Also, O had the lowest SFA content while PO had the highest (*P* < 0.001). For individual FA, oleic acid showed the highest values for O while palmitic acid did for PO (*P* < 0.001). Moreover, O had the lowest content in linoleic acid among dietary treatments (*P* < 0.001). In relation to breast meat, differences were similar to those obtained for abdominal fat pad. Breast meat from animals fed O was the richest in MUFA, while that from animals fed OA was richest in PUFA and that from animals fed PO was richest in SFA (*P* < 0.001). Hence, both O and OA had a higher unsaturated-to-saturated FA (**UFA:SFA**) ratio than PO (*P* < 0.001). In terms of individual FA, palmitic acid showed the highest values for PO, oleic acid did for O and linoleic acid did for OA (*P* < 0.001).

**Table 6:** Fatty acid composition (%) of abdominal fat pad from female broiler chickens according to different dietary fat sources.

	Dietary treatments				
Item	PO	O	OA	SEM^1^	*P*-value
Abdominal fat pad, %	1.31^ab^	1.44^a^	1.25^b^	0.05	0.023
Sum of FA, mg/g	717.80	744.85	708.91	13.28	0.150
SFA	30.73^a^	25.64^c^	27.90^b^	0.50	< 0.001
MUFA	47.38^c^	54.78^a^	49.71^b^	0.64	< 0.001
PUFA	21.89^a^	19.58^b^	22.40^a^	0.53	0.002
UFA:SFA	2.26^c^	2.90^a^	2.60^b^	0.06	< 0.001
n-6:n-3	19.53^b^	17.91^a^	18.82^ab^	0.61	0.049
C16:0	24.44^a^	20.13^c^	21.85^b^	0.48	< 0.001
C16:1	4.00	3.73	3.96	0.18	0.507
C18:0	5.02	4.76	5.11	0.13	0.157
C18:1 n-9	41.44^c^	48.17^a^	43.61^b^	0.58	< 0.001
C18:1 n-7	1.60^b^	1.97^a^	1.74^b^	0.05	< 0.001
C18:2 n-6	20.14^a^	18.21^b^	20.57^a^	0.49	0.005
C18:3 n-3	1.07	1.07	1.13	0.04	0.464
Minor FA	2.21^a^	1.86^b^	2.04^ab^	0.06	0.001

Abbreviations: FA, fatty acids; SFA, saturated fatty acids; MUFA, monounsaturated fatty acids; O, olive pomace oil diet; OA, olive pomace acid oil diet; PO, palm oil diet; PUFA, polyunsaturated fatty acids; SEM, standard error of the mean; UFA, unsaturated fatty acids.

1 n = 8.

a-c Values within a row with different superscripts differ significantly at *P* < 0.05.

**Table 7:** Fatty acid composition (%) of breast meat from female broiler chickens according to different dietary fat sources.

	Dietary treatments				
	PO	O	OA	SEM^1^	*P*-value
Sum of FA, mg/g	13.63	13.53	13.52	1.06	0.994
SFA	32.49^a^	27.03^c^	28.92^b^	0.24	< 0.001
MUFA	41.92^c^	48.47^a^	44.42^b^	0.37	< 0.001
PUFA	25.58^b^	24.50^c^	26.65^a^	0.23	< 0.001
UFA:SFA	3.68^b^	4.89^a^	4.39^a^	0.19	< 0.001
n-6:n-3	14.37^a^	19.94^b^	16.46^a^	0.10	< 0.001
C16:0	23.83^a^	18.85^c^	20.30^b^	0.13	< 0.001
C16:1	3.09^a^	2.50^b^	2.68^b^	0.10	0.002
C18:0	8.07	7.82	8.16	0.19	0.433
C18:1 n-9	36.26^c^	42.84^a^	38.93^b^	0.33	< 0.001
C18:1 n-7	2.18^c^	2.64^a^	2.40^b^	0.06	< 0.001
C18:2 n-6	19.33^b^	18.30^c^	20.24^a^	0.19	< 0.001
C18:3 n-3	0.74^b^	0.82^a^	0.86^a^	0.02	< 0.001
C20:1 n-9	0.39^c^	0.48^a^	0.42^b^	0.01	< 0.001
C20:4 n-6	4.08	3.98	4.11	0.20	0.888
Minor FA	2.04^a^	1.77^b^	1.91^ab^	0.05	0.004

Abbreviations: FA, fatty acids; SFA, saturated fatty acids; MUFA, monounsaturated fatty acids; O, olive pomace oil diet; OA, olive pomace acid oil diet; PO, palm oil diet; PUFA, polyunsaturated fatty acids; SEM, standard error of the mean; UFA, unsaturated fatty acids.

1 n = 8.

a-c Values within a row with different superscripts differ significantly at *P* < 0.05.

## DISCUSSION

### Growth Performance and Feed Intake

The present results show that the inclusion of olive pomace oil, which is rich in monounsaturated FA and mainly composed of triacylglycerols, improved performance parameters and feed efficiency in both growing (from d 22 to 29) and finishing (from d 30 to 39) periods, which resulted in a better FCR when considering each period and the overall trial. As far as we know, no other studies have assessed the effects of olive pomace oil or olive pomace acid oil in broiler chickens. In agreement with the present results, Crespo and Esteve-Garcia (2001) reported an increase in feed efficiency in growing-finishing broilers fed olive oil, which has a similar FA profile to olive pomace oil, compared to those fed tallow, which is rich in SFA. However, Zhang et al. (2013) did not observe differences in broilers fed olive oil compared to those fed tallow. The improved feed efficiency in animals fed O compared to those fed PO may be explained by the higher degree of unsaturation, since many authors have reported that digestibility increases as the degree of unsaturation does (Tancharoenrat et al., 2014; Rodriguez-Sanchez et al., 2019a,^2021^; Jimenez-Moya et al., 2021b). In contrast, OA performed more poorly than O despite having a similar FA composition. This could be related either to the higher FFA or MIU content, or both. It has been described that high FFA content could lead to a lower AME (Wiseman et al., 1992; Powles et al., 1993), which is in agreement with the present results as OA showed a lower AME than O. Similarly, a higher MIU content also dilutes the energy content of the added fat source (Varona et al., 2021a). Hence, the higher OA FFA and MIU contents than O could explain the lower AME values. In turn, this could be related to the higher ADFI found in the finishing period (from 30 to 39 d) for animals fed OA in comparison to O, since broilers tend to vary their feed intake in order to cover their energy requirements (NRC, 1994).

### Digestibility Balance

In the present study, O and OA showed higher FA digestibility than PO, both in AID and ATTD. This was expected because, as mentioned earlier, digestibility increases as the degree of unsaturation does. When comparing FA AID of OA with O, similar results were obtained for total and UFA. However, for SFA and stearic acid, lower AID values were obtained in OA compared to O. The lower digestibility of SFA in OA could be explained by the higher content of FFA. Some authors agree that the explanation for the different fat utilization in diets rich in FFA is found in the absorption processes (Rodriguez-Sanchez et al., 2019a; Jimenez-Moya et al., 2021a). First, due to a lower monoacylglycerol content and bile acid secretion in the duodenum (Sklan, 1979; Atteh and Leeson, 1985), which are considered essential for the formation of mixed micelles and hence the absorption of lipolysis end-products (Krogdahl, 1985; Ravindran et al., 2016). On the other hand, FFA can interact with ionized minerals, forming insoluble soaps that are unavailable for absorption (Small 1991; Jimenez-Moya et al., 2021a). Concretely, and in accordance with the present results, this effect has been found to be much more pronounced in SFA rather than UFA (Atteh and Leeson, 1985; Wiseman and Salvador, 1991). In agreement with this, other studies in broiler chickens have found that the ileal digestibility of SFA decreases as dietary FFA content increases (Rodriguez-Sanchez et al., 2019a). However, although absorption of SFA could have been compromised by the presence of FFA in OA, the values obtained for these animals were much higher than those obtained for PO. These results suggest that the saturation degree had more influence on FA digestibility than the dietary FFA content did, which is in agreement with other previous studies (Vilarrasa et al., 2015; Rodriguez-Sanchez et al., 2019a; Jimenez-Moya et al., 2021a; Rodriguez-Sanchez et al., 2021).

The present study showed that when acid oils are included in growing-finishing diets (from 22 to 39 d) only SFA is affected, and no changes in TFA, MUFA or PUFA AID are observed, which may be explained by the fact that saturated FFA are more prone than unsaturated FFA to form insoluble soaps. In agreement with the present results, recent studies showed that adding a moderate content of dietary FFA does not negatively affect TFA digestibility. Rodriguez-Sanchez et al. (2021) and Jimenez-Moya et al. (2021a) did not find negative effect on the digestibility of TFA in growing-finishing broiler chickens that were fed diets containing up to 35% and 30% of FFA, respectively. Furthermore, the use of olive pomace acid oil (38.6% of dietary FFA) in growing-finishing pigs did not affect the digestibility of TFA compared to olive pomace oil (Verge-Mèrida et al., 2021). In fact, other authors have found that the effect of dietary FFA is limited to SFA, and especially in young animals, that have lower production and secretion of bile acids, which hinders their dietary fat assimilation (Wiseman et al., 1991; Rodriguez-Sanchez et al., 2019a; Jimenez-Moya et al., 2021a). Hence, in agreement with previous studies, the present results suggest that acid oils could be included in growing-finishing diets without major impairment of FA digestibility, at least when dietary FFA content does not exceed 30% and the dietary UFA:SFA ratio is above 3.46.

In general, similar values were obtained for ATTD to those obtained for AID. This was expected since the absorption of fat is practically negligible in the hindgut of poultry (Renner, 1965; Ravindran et al., 2016). However, in contrast to what was observed in the AID of FA, values for ATTD were lower in OA when compared to O for most of the FA, with the exception of PUFA. This is in accordance with other studies that described how high dietary FFA has negative effects on fat digestibility measured at fecal level (Blanch et al., 1995,^1996^; Vilà and Esteve-Garcia, 1996; Wiseman et al., 1998). This effect could be caused by bacterial activity, mainly in the cecum. In this regard, bacteria biohydrogenation of oleic, linoleic, and linolenic acids would convert them into stearic acid and other FA that originate from this activity (Duran-Montgé et al., 2007; Rodriguez-Sanchez et al., 2019a, 2021), which therefore affects the ATTD values, especially those of SFA and MUFA. Previous studies (Rodriguez-Sanchez et al. 2019a; Rodriguez-Sanchez et al. 2021) found increasing concentrations of FA produced by bacterial activity (capric acid, C10:0; margaric acid, C17:0; *trans* C18:1; and vaccenic acid, C18:1 n-7) as dietary FFA content increased, suggesting that the higher the dietary FFA content, the greater the bacterial activity. These results indicate that AID data are more accurate and should be used instead of ATTD data for FA digestibility, since the microbial effect and other confounding factors such as endogenous losses are thus avoided (Stein, 2017).

### Fatty Acid Composition of Abdominal Fat and Breast Meat

In the present study, animals fed O showed a higher abdominal fat deposition than those fed OA. In fact, olive pomace acid oil used in this study had a higher content in PUFA, which previous studies have associated with a decrease in abdominal fat deposition (Crespo and Esteve-Garcia, 2002a,b; Ferrini et al., 2008; Vilarrasa et al., 2015). Preferential β-oxidation of PUFA with respect to SFA or MUFA and a decreased rate of FA synthesis could explain this (Crespo and Esteve-Garcia, 2002c). Moreover, dietary PUFA seem to reduce serum levels of insulin and of very low density lipoproteins (Crespo and Esteve-Garcia, 2003), which also limits fat deposition.

The FA profile of both abdominal fat pad and breast meat reflected that of the diet, depending on the added fat source, which is in agreement with the results reported in the literature (Ferrini et al., 2008; Vilarrasa et al., 2015; Skřivan et al., 2018; Viñado et al., 2020). Concretely, in O and OA treatments, SFA were reduced in both tissues, increasing the content in MUFA and the UFA:SFA ratio when compared to PO. The reduction of SFA in breast meat contributes to the global trend towards producing healthy meat products, since consumption of saturated fat have been related to many health concerns (Islam et al., 2019; López-Pedrouso et al., 2021).

In conclusion, the inclusion of olive pomace oil at 6% in growing finishing broiler diets achieved better performance, feed efficiency, and digestibility compared to a conventional source such as palm oil. On the other hand, when olive pomace acid oil (fat by-product rich in FFA) is used instead of palm oil, no negative effect was observed in performance or feed efficiency. However, olive pomace acid oil showed lower digestibility of SFA than olive pomace oil, although no changes in TFA, MUFA, or PUFA ileal digestibility were observed. Additionally, the inclusion of olive pomace oil or olive pomace acid oil leads to a reduction in saturated fatty acids in both abdominal fat and breast meat compared to palm oil. Hence, the present results suggest that olive pomace oil and acid oil are interesting sources for inclusion in growing-finishing broiler chicken diets, which may potentially reduce feeding costs and contribute to more efficient production and the circular economy.
